# Predictors of Mortality in Acute Myocardial Infarction: Insights From the Healthcare Cost and Utilization Project (HCUP) Nationwide Readmission Database

**DOI:** 10.7759/cureus.83675

**Published:** 2025-05-07

**Authors:** Priji Prasad Jalaja, Dheeraj Kommineni, Aashish Mishra, Ramakrishna Tumati, Chrishanti Anna Joseph, Rana Veer Samara Sihman Bharattej Rupavath

**Affiliations:** 1 Surgery, Emory University School of Medicine, Atlanta, USA; 2 Systems Analytics, Hanker Systems, Chantilly, USA; 3 Computer and Information Science, Eastern Kentucky University, Richmond, USA; 4 Software Application and Engineering, Intel, Beaverton, USA; 5 Anesthesiology and Perioperative Medicine, University of Pittsburgh, Pittsburgh, USA; 6 Business Administration, National Louis University, Tampa, USA

**Keywords:** acute myocardial infarction, covid-19, healthcare cost and utilization project, hospitalization outcomes, nationwide readmission database

## Abstract

Background

Acute myocardial infarction (AMI) remains a significant concern for morbidity and mortality globally. Understanding AMI-related mortality predictors can help mitigate risks and improve patient outcomes. This study was conducted to evaluate demographic, clinical, and institutional factors associated with in-hospital mortality among patients admitted for AMI.

Methods

A retrospective analysis was conducted using the 2020 Nationwide Readmission Database (NRD). Adult patients (≥18 years) with a primary diagnosis of AMI were included. COVID-19 co-infection was identified via secondary diagnoses. Exclusion criteria comprised missing mortality data. Patient demographics, comorbidities, and hospital characteristics were analyzed. A survey-weighted multivariable logistic regression, incorporating discharge weights, stratification, and clustering, was used to estimate odds ratios (OR) for 30-day all-cause mortality (death during index admission or within 30-day unplanned readmission), adjusting for the above covariates, including COVID-19 status.

Results

Of 1,193,046 AMI admissions, 127,128 (10.7%) died within 30 days. Median age was 75 (65-83) years among non-survivors compared to 69 (59-79) years in survivors. Key multivariable predictors of higher mortality included All Patients Refined Diagnosis-Related Group (APR-DRG) “extreme” severity (OR 3.82, p < 0.001), cardiac arrest (OR 7.03, p < 0.001), cardiogenic shock (OR 1.52, p < 0.001), acute kidney injury (OR 1.82, p < 0.001), and COVID-19 co-infection (OR 2.51, p < 0.001). Institutional factors such as treatment at micropolitan hospitals (OR 1.72, p < 0.001) and self-pay status (OR 1.24, p < 0.001) also increased risk, with additional categories for Medicare, Medicaid, and private payers examined.

Conclusion

In conclusion, predictors of mortality in AMI patients include baseline characteristics such as age and gender, comorbidities like heart failure and cardiac arrest, socioeconomic factors, and the impact of COVID-19. Future studies should explore patient-level race/ethnicity and education data, assess vaccination effects, and develop targeted interventions to reduce these disparities.

## Introduction

Acute myocardial infarction (AMI) remains a significant contributor to global morbidity and mortality. Globally, three million people are affected by AMI, with one million deaths reported in the United States annually [1]. AMI includes ST-segment elevation myocardial infarction (STEMI) and non-ST-elevation myocardial infarction (NSTEMI) [2]. Although management strategies have improved significantly over the years, including percutaneous coronary intervention (PCI) and pharmacological therapies, the mortality risk associated with AMI still remains high. Recent evidence suggests that prolonged (>1 year) beta-blocker therapy may not provide additional mortality benefit in patients without reduced ejection fraction. Identification of predictors of mortality in AMI patients is crucial for optimizing management protocols for them [3]. Several studies have shown various clinical, demographic, and biochemical factors that contribute to mortality risks in AMI [4,5]. For example, advanced age, reduced left ventricular ejection fraction (LVEF), and the presence of comorbidities such as diabetes mellitus (DM) and heart failure are consistently associated with poorer prognoses [6]. LVEF has particularly emerged as a critical determinant, with lower LVEF being consistently associated with increased mortality risk, especially in the early post-infarction period [1]. Comorbidities, including DM and chronic kidney disease, further complicate clinical presentation and exacerbate patient outcomes [1,6]. Anatomic disease burden, such as severe multivessel or left main coronary artery disease (CAD), and modifiable risk factors both drive AMI incidence and influence post-AMI mortality.

Another factor is CAD, the main underlying condition for AMI, which is influenced by modifiable risk factors like smoking, hypertension, sedentary lifestyle, and DM, and non-modifiable factors including age, sex, race, and family history [7]. Thus, all these factors have the potential to influence AMI. The risk of AMI further increases in populations that have a prevalence of multiple risk factors like DM, low physical activity, and hypertension [8]. Moreover, emerging data indicate that concurrent COVID-19 infection may exacerbate endothelial dysfunction, promote plaque instability, and amplify inflammatory responses, thereby increasing AMI severity and mortality. Furthermore, recent research has shown the potential role of novel biomarkers and advanced imaging techniques in identifying predictors of AMI-related outcomes. For instance, troponin elevations exceeding five times the upper reference limit and high-sensitivity C-reactive protein levels above 3 mg/L have been associated with increased AMI-related mortality risk [9]. With the increasing incorporation of artificial intelligence in the medical field, the use of machine learning algorithms has also increased to analyze large datasets, identifying complex patterns that may predict mortality more accurately than conventional methods. Several well-validated risk stratification tools, such as the Thrombolysis in Myocardial Infarction (TIMI) and Global Registry of Acute Coronary Events (GRACE) scores, can estimate short- and long-term mortality risk [9]. AMI-related mortality is also influenced by timely intervention because factors such as the speed of reperfusion therapy and the presence of cardiogenic shock at presentation are critical in determining patient outcomes [10]. Cardiogenic shock significantly increases the risk of death and requires prompt intervention. Due to these complexities, it is important to understand the predictors of mortality in AMI. These findings can help clinicians make informed decisions [2].

Previously, several studies have identified predictors of mortality in AMI patients [11-13]. However, there is still a gap in the understanding of institutional and socioeconomic factors contributing to AMI mortality. Social determinants, including educational attainment, neighborhood deprivation, and income inequality, are increasingly recognized as powerful drivers of cardiovascular outcomes. Furthermore, the impact of differences in hospital settings (e.g., urban vs. micropolitan hospitals) and insurance status is also less understood. Therefore, this study was carried out to address the high mortality rates associated with AMI by identifying predictors of mortality.

## Materials and methods

Study design and population

This study was conducted at the Cryptch IT Solutions, Jabalpur, Madhya Pradesh, India. A retrospective analysis was conducted using de-identified patient data from the 2020 Nationwide Readmission Database (NRD). We included all patients ≥ 18 years admitted with a primary diagnosis of AMI. COVID-19 status during the index admission was not an inclusion requirement, but was ascertained as a secondary diagnosis. Index admissions were identified based on COVID-19 and AMI diagnosis codes. As the study utilized de-identified data, it was exempt from Institutional Review Board (IRB) approval. While the NRD is publicly available, access requires an application and adherence to data use agreements.

Data source

The NRD provided data from January 1, 2020, to December 31, 2020. The NRD is the largest all-patient, all-payer inpatient database in the United States, created and maintained by the Agency for Healthcare Research and Quality for the HCUP. It includes hospitalization data from nonfederal hospitals across 31 geographically diverse states, representing 60.8% of all U.S. hospitalizations and 62.2% of the population. These coverage rates are consistent with previous years, though state participation may introduce selection biases. The NRD allows analysis at both the hospital and patient levels, with up to 40 discharge diagnoses and 25 procedures recorded using the International Classification of Diseases, 10th Revision, Clinical Modification (ICD-10-CM) [14]. Hospitals are categorized by geographic region, teaching status, bed count, ownership, and urban/rural location. The NRD enables weighted analysis to estimate nationwide hospitalizations for a given year. To ensure diagnostic accuracy, our ICD-10-CM code lists were drawn from the Agency for Healthcare Research and Quality (AHRQ) HCUP Clinical Classifications Software (CCS) definitions for AMI and COVID-19. Furthermore, it was reviewed and finalized by two board-certified cardiologists and one certified medical coder. Patients older than or equal to 18 years old with a primary diagnosis of AMI based on ICD-10-CM codes were eligible to participate. Among the exclusion criteria were patients under 18 years old, and death data missing from the index admission.

Covariates

Patient demographics for the index admission included gender, age, insurance coverage, median household income, functional status impairment (e.g., mobility limitations or organ dysfunction), and discharge disposition. ICD-10-CM codes were used to identify comorbidities, which were analyzed individually rather than using a specific comorbidity index such as Elixhauser or Charlson. Hospital characteristics, including bed size, ownership (private vs. government), designation (large, small, micropolitan, non-urban), and teaching status, were also collected. ICD-10 procedure codes identified interventions performed during hospitalization. The All Patients Refined Diagnosis-Related Group (APR-DRG) was used to assess disease severity. The primary outcome was all-cause mortality, defined as death during the index AMI hospitalization or during any unplanned 30-day readmission. The secondary outcome was any unplanned 30-day hospital readmission following discharge from the index AMI admission.

Statistical analysis

IBM SPSS Statistics for Windows, Version 23 (Released 2015; IBM Corp., Armonk, New York, United States) was used for all statistical analyses. Before selecting a non-parametric test, normality assumptions were assessed using the Shapiro-Wilk test. The Pearson chi-square test was applied to categorical variables, while the Mann-Whitney U test was used for continuous variables, with no mortality as the reference group, to compare baseline characteristics such as gender, age, weekend versus weekday admissions, household income, payer status, functional status impairment, and likelihood of dying. Multivariable logistic regression was conducted to examine clinical factors associated with 30-day readmission, adjusting for potential confounders such as disease severity (APR-DRG) and pre-existing conditions (individual comorbidities). Each candidate covariate (demographics, payer, income quartile, APR-DRG severity, COVID-19 status, individual comorbidities, functional impairment, hospital factors, and key procedural variables) was tested in univariate models; those with p < 0.10 were considered for multivariable analysis. Additionally, multiple logistic regression models were used to identify independent predictors of mortality, analyzing deaths occurring both during the initial hospitalization and within the readmission period. Results were reported as odds ratios (OR) with 95% confidence intervals (CI).

Patient and public involvement

This study utilized the NRD, a publicly accessible all-payer inpatient healthcare readmission database in the United States, derived from the AHRQ HCUP. As the NRD contains de-identified data, no direct patient involvement, patient-reported outcomes, or perspectives were included in this study. Consequently, we were not able to capture patient-reported outcomes, qualitative insights, or individual perceptions of care, particularly regarding social support, health literacy, and other social determinants of health that may modify the association between socioeconomic or institutional factors and post-AMI outcomes. We acknowledge that omission of these patient perspectives represents a limitation.

Data availability statement

The Nationwide Readmissions Database (NRD) is available from the AHRQ HCUP after completion of a formal application, purchase of the data, execution of a data use agreement, and successful completion of HCUP’s data use training. Researchers may request access via the AHRQ HCUP website.

## Results

Baseline characteristics

A total of 1,193,046 participants were included in the analysis, with non-survivors (10.7%, n=127,128) and survivors (89.3%, n=1,065,918). The median age of the mortality group was 75 (65-83) years, while it was 69 (59-79) years in the non-mortality group. Women constituted 40.35% of the mortality cohort. Weekend admissions were more frequent among the mortality group (26.6%) compared to the overall population (26.3%) (p=0.009). Medicare coverage was more common among mortality cases (74.5% vs. 64.2% overall, p < 0.001), while private insurance was less frequent (11.8% vs. 18.9%, p < 0.001). Hospitalization costs and length of stay (LOS) were higher in the mortality group, with median hospitalization costs at $98,429 compared to $72,360 in the overall cohort. Regarding income quartiles, patients in the lowest income quartile (0-25th percentile) represented the largest subgroup in both mortality (31.9%) and overall (31.3%) groups (p < 0.001). The likelihood of dying (APR-DRG) was extreme in 90.8% of mortality cases compared to 40.3% in the overall cohort (p < 0.001). Hospitalizations at large hospitals (55.3%) and private non-profit hospitals (73.7%) were predominant across all groups (Table 1).

**Table 1:** Baseline characteristics during index admission for AMI LOS: length of stay; IQR: interquartile range; APR-DRG: All Patients Refined Diagnosis-Related Group; AMI: acute myocardial infarction

Characteristics	No Mortality (n=1065918; 89.3%)	Mortality (n=127128; 10.7%)	Overall (n=1193046)	P-value
Age (in years), median (IQR)	69 (59-79)	75 (65-83)	70 (60-79)	
Women	(430005; 40.3%)	(53449; 42.0%)	(483454; 40.5%)	< .001
Elective	(32523; 3.1%)	(3755; 3.0%)	(36278; 3.0%)	0.055
Weekend admission	(279570; 26.2%)	(33775; 26.6%)	(313345; 26.3%)	0.009
Insurance status	-	-	-	-
Medicare	(670609; 63.0%)	(94531; 74.5%)	(765140; 64.2%)	< .001
Medicaid	(109083; 10.3%)	(10098; 8.0%)	(119181; 10.0%)	< .001
Private	(209954; 19.7%)	(15025; 11.8%)	(224979; 18.9%)	< .001
Self-pay	(36861; 3.5%)	(2938; 2.3%)	(39799; 3.3%)	< .001
No charge	(4726; 0.4%)	(184; 0.1%)	(4910; 0.4%)	< .001
Other	(32938; 3.1%)	(4070; 3.2%)	(37008; 3.1%)	< .001
Cost of hospitalization in US$, median (IQR)	74176.00	98429.00	72360.00	-
LOS, median (IQR)	4	5	4	-
Quartile of median household income	-	-	-	-
0-25^th^	(328746; 31.2%)	(39993; 31.9%)	(368739; 31.3%)	< .001
26th-50^th^	(314720; 29.9%)	(37530; 29.9%)	(352250; 29.9%)	< .001
51st-75^th^	(234910; 22.3%)	(27197; 21.7%)	(262107; 22.2%)	< .001
76th-100^th^	(174386; 16.6%)	(20761; 16.5%)	(195147; 16.6%)	< .001
APR-DRG, likelihood of dying	-	-	-	-
Minor	(155955; 14.6%)	(399; 0.3%)	(156354; 13.1%)	< .001
Moderate	(199438; 18.7%)	(1276; 1.0%)	(200714; 16.8%)	< .001
Major	(345186; 32.4%)	(9969; 7.8%)	(355155; 29.8%)	< .001
Extreme	(365320; 34.3%)	(115472; 90.8%)	(480792; 40.3%)	< .001
APR-DRG, loss of function	-	-	-	-
Minor (includes cases with no comorbidity or complications)	(146571; 13.8%)	(527; 0.4%)	(147098; 12.3%)	< .001
Moderate	(331898; 31.1%)	(2916; 2.3%)	(334814; 28.1%)	< .001
Major	(323487; 30.3%)	(17893; 14.1%)	(341380; 28.6%)	< .001
Extreme	(263943; 24.8%)	(105781; 83.2%)	(369724; 31.0)	< .001
Hospital bed size	-	-	-	-
Small	(182128;17.1%)	(21119; 16.6%)	(203247; 17.0%)	< .001
Medium	(295688;27.7%)	(34497; 27.1%)	(330185; 27.7%)	< .001
Large	(588101;55.2%)	(71512; 56.3%)	(659613; 55.3%)	< .001
Control/ownership of the hospital	-	-	-	
Government	(107012; 10.0%)	14036; 11.0%)	(121048; 10.1%)	
Private non-profit	(786404; 73.8%)	(92409; 72.7%)	(878813; 73.7%)	< .001
Private for-profit	(172501; 16.2%)	(20683; 16.3%)	(193184; 16.2%)	< .001
Hospital designation	-	-	-	-
Large metropolitan, ≥1 million residents	(542561;50.9%)	(68615; 54.0%)	(611176; 51.2%)	< .001
Small metropolitan, ≤1 million residents	(441711; 41.4%)	(48684; 38.3%)	(490395; 41.1%)	< .001
Micropolitan	(70081; 6.6%)	(7923; 6.2%)	(78004; 6.5%)	< .001
Non-urban residual	(11564; 1.1%)	(1905; 1.5%)	(13469; 1.1%)	< .001
Hospital teaching status	-	-	-	-
Metropolitan, non-teaching	(196925; 18.5%)	(22143; 17.4%)	(219068; 18.4%)	< .001
Metropolitan, teaching	(787347; 73.9%)	(95156; 74.9%)	(882503; 74.0%)	< .001
Non-metropolitan	(81645; 7.7%)	(9828; 7.7%)	(91473; 7.7%)	< .001
Discharge location	-	-	-	-
Home self-care	(611833; 57.4%)	(0; 0.0%)	(611833; 51.3%)	< .001
Home health care	(24312; 2.3%)	(0; 0.0%)	(24312; 2.0%)	< .001
Transfer to a short-term hospital	(183926; 17.3%)	(0; 0.0%)	(183926; 15.4%)	< .001
Against medical advice	(224423; 21.1%)	(0; 0.0%)	(224423; 18.8%)	< .001

Comorbidities and procedure-related factors

Patients in the mortality group had significantly higher rates of comorbidities and complications compared to the non-mortality group (p < 0.001). Heart failure prevalence was 55.0% in the mortality group compared to 47.1% overall. Cardiac arrest was significantly more common in the mortality group (24.1%) compared to 4.9% overall. Shock was present in 28.7% of the mortality group, markedly higher than 8.3% overall. Respiratory failure prevalence was 79.5% in the mortality group compared to 32.0% overall. Acute kidney injury (AKI) was observed in 67.1% of the mortality group compared to 35.5% overall. Fluid and electrolyte disorders were found in 75.6% of the mortality group compared to 44.6% overall. However, tobacco use and alcohol abuse were less common among mortality cases (0.0% and 3.4%, respectively) (Table 2).

**Table 2:** Comorbidities and procedure-related factors during index admission for AMI AKI: acute kidney injury; COPD: chronic obstructive pulmonary disease; CKD: chronic kidney disease; AMI: acute myocardial infarction; PAD: peripheral arterial disease; CVA: cerebrovascular accident; TIA: transient ischemic attack; PCI: percutaneous coronary intervention; CABG: coronary artery bypass grafting; ALL: acute lymphoblastic leukemia; GI: gastrointestinal; COVID-19: coronavirus disease 2019

Comorbidities	No Mortality (n= 1065918; 89.3%)	Mortality (n=127128; 10.7%)	Overall (n=1193046)	P-value
Cardiac arrest	(28307; 2.7%)	(30656; 24.1%)	(58963; 4.9%)	< .001
Heart failure	(492172; 46.2%)	(69877; 55.0%)	(562049; 47.1%)	< .001
Coronary atherosclerosis	(738065; 69.2%)	(61965; 48.7%)	(800030; 67.1%)	< .001
Shock	(63126; 5.9%)	(36486; 28.7%)	(99612; 8.3%)	< .001
Cardiac dysrhythmias	(376017; 35.3%)	(62831; 49.4%)	(438848; 36.8%)	< .001
AKI	(338088; 31.7%)	(85326; 67.1%)	(423414; 35.5%)	< .001
COPD	(231239; 21.7%)	(31392; 24.7%)	(262631; 22.0%)	< .001
CKD	(342441; 32.1%)	(50592; 39.8%)	(393033; 32.9%)	< .001
Respiratory failure	(281136; 26.4%)	(101094; 79.5%)	(382230; 32.0%)	< .001
Fluid and electrolyte disorders	(436316; 40.9%)	(96124; 75.6%)	(532440; 44.6%)	< .001
Cardiogenic shock	(45201; 4.2%)	(27238; 21.4%)	(72439; 6.1%)	< .001
Tobacco use	(1674; 0.2%)	(59; 0.0%)	(1733; 0.1%)	< .001
Alcohol abuse	(42995; 4.0%)	(4356; 3.4%)	(47351; 4.0%)	< .001
Lipid disorders	(678433; 63.6%)	(57280; 45.1%)	(735713; 61.7%)	< .001
Hypertension	(418005; 39.2%)	(28579; 22.5%)	(446584; 37.4%)	< .001
Diabetes	(451108; 42.3%)	(54300; 42.7%)	(505408; 42.4%)	0.007
Obesity	(224303; 21.0%)	(17887; 14.1%)	(242190; 20.3%)	< .001
Valvular heart disease	(104406; 9.8%)	(11345; 8.9%)	(115751; 9.7%)	< .001
PAD	(80634; 7.6%)	(9997; 7.9%)	(90631; 7.6%)	< .001
GI bleed	(41632; 3.9%)	(12082; 9.5%)	(53714; 4.5%)	< .001
Liver failure	(22337; 2.1%)	(20737; 16.3%)	(43074; 3.6%)	< .001
Thyroid disorders	(158551; 14.9%)	(18059; 14.2%)	(176610; 14.8%)	< .001
Anemia	(93163; 8.7%)	(15193; 12.0%)	(108356; 9.1%)	< .001
Coagulation disorders	(102462; 9.6%)	(30229; 23.8%)	(132691; 11.1%)	< .001
Cancer	(89285; 8.4%)	(16745; 13.2%)	(106030; 8.9%)	< .001
Depression	(120390; 11.3%)	(10860; 8.5%)	(131250; 11.0%)	< .001
Dementia and neurocognitive disorders	(98715; 9.3%)	(20047; 15.8%)	(118762; 10.0%)	< .001
Complications of the device	(58215; 5.5%)	(5784; 4.5%)	(63999; 5.4%)	< .001
Pneumonia	(159782; 15.0%)	(54178; 42.6%)	(213960; 17.9%)	< .001
CVA	(33141;3.1%)	(9835;7.7%)	(42976; 3.6%)	< .001
TIA	(4362; 0.4%)	(327; 0.3%)	(4689; 0.4%)	< .001
History of PCI	(165005; 15.5%)	(11197; 8.8%)	(176202; 14.8%)	< .001
History of CABG	(26924; 2.5%)	(1834; 1.4%)	(28758; 2.4%)	< .001
Vasopressors	(24272; 2.3%)	(18896; 14.9%)	(43168; 3.6%)	< .001
Pulmonary circulatory disorders	(94048; 8.8%)	(13523; 10.6%)	(107571; 9.0%)	< .001
In hospital bleeding	(4404; 0.4%)	(675; 0.5%)	(5079; 0.4%)	< .001
In hospital vascular complications	(1761; 0.2%)	(141; 0.1%)	(1902; 0.2%)	< .001
Conduction disorder	(125870; 11.8%)	(16336; 12.9%)	(142206; 11.9%)	< .001
PCI ALL	(325278; 30.5%)	(14236; 11.2%)	(339514; 28.5%)	< .001
CABG during admission	(60598; 5.7%)	(2559; 2.0%)	(63157; 5.3%)	< .001
Impella	(7802; 0.7%)	(4832; 3.8%)	(12634; 1.1%)	< .001
COVID-19	(43284; 4.1%)	(25551; 20.1%)	(68835; 5.8%)	< .001

Table 3 shows multivariable logistic regression analysis for predicting mortality. Younger age groups had significantly lower odds of mortality compared to patients aged 75 years and older. Female patients had a lower likelihood of mortality compared to males (OR=0.971, p < 0.001). Regarding insurance status, self-pay patients (OR=1.236, p < 0.001) and those with Medicare (OR=1.087, p < 0.001) had higher mortality risk compared to Medicaid beneficiaries, whereas patients with no-charge status had a lower risk (OR=0.754, p=0.002). Clinical severity played a major role in predicting mortality. Patients classified as having an “extreme” likelihood of dying had the highest odds of mortality (OR=3.816, p < 0.001). Hospital-related factors also influenced mortality outcomes. Medium-sized hospitals were associated with a slight increase in mortality risk (OR=1.023, p=0.045). Private non-profit (OR=0.926, p < 0.001) and private for-profit (OR=0.874, p < 0.001) hospitals had lower mortality risks compared to government-owned hospitals. Several comorbidities significantly impacted mortality risk. Patients with cardiac arrest (OR=7.033, p < 0.001), respiratory failure (OR=1.906, p < 0.001), liver failure (OR=2.174, p < 0.001), shock (OR=1.520, p < 0.001), and gastrointestinal bleeding (OR=1.303, p < 0.001) had the highest risk of mortality. Cancer (OR=1.259, p < 0.001) and dementia (OR=1.027, p < 0.001) also contributed to increased mortality risk. Procedural and disease-specific factors further influenced mortality. Patients requiring vasopressors (OR=2.123, p < 0.001) or an Impella device (OR=2.587, p < 0.001) had significantly higher odds of death. Furthermore, COVID-19 was a major predictor, nearly doubling the odds of mortality (OR=2.505, p < 0.001).

**Table 3:** Multivariable logistic regression analysis for predicting mortality AKI: acute kidney injury; COPD: chronic obstructive pulmonary disease; CKD: chronic kidney disease; AMI: acute myocardial infarction; PAD: peripheral arterial disease; CVA: cerebrovascular accident; TIA: transient ischemic attack; PCI: percutaneous coronary intervention; CABG: coronary artery bypass grafting; ALL: acute lymphoblastic leukemia; GI: gastrointestinal; COVID-19: coronavirus disease 2019

Variable	OR (95% CI)	P-value
Age	-	-
≥75	1 (reference)	
18-44	0.259 (0.247-0.272)	< .001
45-64	0.436 (0.425-0.447)	< .001
65-74	0.634 (0.623-0.646)	< .001
Sex	-	-
Male	1 (reference)	
Female	0.971 (0.956-0.986)	< .001
Insurance status	-	-
Medicaid	1 (reference)	
Medicare	1.087 (1.053-1.122)	< .001
Private insurance	0.972 (0.940-1.005)	0.095
Self-pay	1.236 (1.169-1.307)	< .001
No charge	0.754 (0.631-0.902)	0.002
Other	1.205 (1.146-1.267)	< .001
Likelihood of dying	-	-
Minor	40.519 (17.191-95.503)	< .001
Moderate	1.635 (1.440-1.857)	< .001
Major	2.443 (2.150-2.775)	< .001
Extreme	3.816 (3.349-4.347)	< .001
Severity of illness	-	-
Minor, includes cases with no morbidity or complications	1 (reference)	
Moderate	0.915 (0.818-1.022)	0.117
Major	1.875 (1.672-2.104)	< .001
Extreme	4.063 (3.614-4.568)	< .001
Hospital bed size	-	-
Small	1 (reference)	-
Medium	1.023 (1.000-1.046)	0.045
Large	1.006 (0.985-1.026)	0.589
Control/ownership of the hospital	-	-
Government	1 (reference)	
Private non-profit	0.926 (0.904-0.948)	< .001
Private for profit	0.874 (0.849-0.899)	< .001
Hospital designation	-	-
Large metropolitan, ≥1 million residents	1.066 (1.050-1.083)	< .001
Small metropolitan, ≤1 million residents	1.134 (1.099-1.169)	< .001
Micropolitan	1.721 (1.622-1.827)	< .001
Comorbidities	-	-
Cardiac arrest	7.033 (6.874-7.196)	< .001
Heart failure	0.629 (0.619-0.640)	< .001
Coronary atherosclerosis	0.867 (0.852-0.881)	< .001
Shock	1.520 (1.471-1.569)	< .001
Cardiac dysrhythmias	1.086 (1.070-1.102)	< .001
AKI	1.406 (1.384-1.429)	< .001
COPD	0.900 (0.884-0.915)	< .001
CKD	0.935 (0.920-0.950)	< .001
Respiratory failure	1.906 (1.866-1.946)	< .001
Fluid and electrolyte disorders	1.277 (1.256-1.299)	< .001
Cardiogenic shock	1.661 (1.601-1.723)	< .001
Tobacco use	0.366 (0.271-0.493)	< .001
Alcohol abuse	0.716 (0.688-0.745)	< .001
Lipid disorders	0.800 (0.788-0.813)	< .001
Hypertension	0.762 (0.747-0.777)	< .001
Diabetes	1.034 (1.019-1.051)	< .001
Obesity	0.768 (0.752-0.784)	< .001
Valvular heart disease	0.946 (0.923-0.970)	< .001
PAD	1.083 (1.055-1.112)	< .001
GI bleed	1.303 (1.267-1.340)	< .001
Liver failure	2.174 (2.120-2.230)	< .001
Thyroid disorders	0.945 (0.926-0.965)	< .001
Anemia	0.704 (0.722-1.256)	< .001
Coagulation disorders	1.235 (0.687-1.258)	< .001
Cancer	1.259 (1.232-1.286)	< .001
Depression	0.827 (0.807-0.847)	< .001
Dementia and neurocognitive disorders	1.027 (1.006-1.049)	< .001
Complications of the device	1.089 (1.051-1.129)	< .001
Pneumonia	1.128 (1.109-1.148)	< .001
CVA	1.583 (1.538-1.629)	< .001
TIA	0.770 (0.675-0.878)	< .001
History of PCI	0.945 (0.922-0.970)	< .001
History of CABG	0.960 (0.905-1.019)	0.178
Vasopressors	2.123 (2.070-2.177)	< .001
Pulmonary circulatory disorders	0.899 (0.878-0.920)	< .001
In hospital bleeding	1.020 (0.915-1.137)	0.719
In hospital vascular complications	0.593 (0.485-0.726)	< .001
Conduction disorder	0.924 (0.904-0.944)	< .001
PCI ALL	0.453 (0.441-0.465)	< .001
CABG during admission	0.404 (0.384-0.424)	< .001
Impella	2.587 (2.459-2.723)	< .001
COVID-19	2.505 (2.449-2.562)	< .001

## Discussion

This study aimed to identify predictors of mortality in AMI patients. The findings of the present study showed that age, sex, insurance status, severity of illness, ownership of hospital, and comorbidities showed significant differences between survivors and non-survivors. Younger age groups were independently protective compared to older patients. Furthermore, patients aged ≥75 years had markedly higher odds of dying compared to younger cohorts, reinforcing age as a pivotal factor in AMI outcomes. This finding aligns with existing literature that suggests older age is a well-established risk factor for increased mortality following AMI [15]. Even the incidence of AMI is unusually higher in older individuals compared to younger individuals [16]. Similarly, Medicare coverage largely overlaps with age ≥65, suggesting its association with higher mortality may reflect age confounding rather than an independent payer effect.

Similarly, gender-related disparities were also observed in our study, with males having a significantly higher risk of mortality compared to females. Previously, studies have reported a higher risk of mortality in young females compared to young males hospitalized with AMI [17,18]. The risk of mortality is particularly higher in women during short-term follow-up duration [19]. A systematic review by Bucholz et al. reported that females have a higher risk of mortality with AMI compared to males during a five- to 10-year period [20]. However, this may not be the case in older individuals. For example, Song et al., in their study, reported that older women, with ages over 65 years, had 20.4% less risk of mortality compared to males in the same age group; however, the females aged less than 65 years had an 84.3% higher rate of mortality compared to males [21]. As in our study, the median age was above 65 years, which may explain the higher mortality rate observed in males compared to females.

Another interesting finding of the study was that weekend admissions were significantly more common in patients who died compared to survivors. This finding raises questions about potential disparities in care quality based on admission timing. A study that assessed 3,789,917 admissions reported that patients with serious conditions, if admitted during weekends, have a higher risk of mortality compared to admissions on weekdays [22]. However, in the present study, this may reflect higher baseline acuity on weekends (e.g., unwitnessed arrests) rather than a systemic “weekend effect.” Future work should adjust for initial presentation severity to clarify this relationship. Furthermore, the predominance of Medicare patients in the mortality group (74.5%) compared to private insurance holders (11.8%) underscores the need to address the healthcare needs of older populations, particularly those reliant on public insurance programs [23]. Economic implications were evident, as patients in the mortality group incurred higher hospitalization costs (US$98,429 vs. US$74,176) and longer lengths of stay (five days vs. four days). These findings suggest that higher resource utilization is associated with poorer outcomes, which emphasizes the importance of efficient care delivery to reduce costs while improving survival rates [24].

In the present study, cardiac arrest was identified as the most significant predictor for mortality in AMI. This aligns with previous studies that identify heart failure as a major predictor of mortality in AMI patients [25]. Ornato et al. reported that cardiac arrest was 2.8 times higher in patients with AMI in their study [26]. Respirator failure was also observed as a significant predictor for mortality in the present study. According to Vallabhajosyula et al., respiratory failure was associated with a 1.7- to 2.2-fold higher risk of in-hospital mortality in patients with AMI [27]. Similarly, liver failure was associated with an increased risk of mortality. Munguti et al. have reported that patients with non-alcoholic fatty liver disease, if admitted with AMI, are more likely to die in hospital [28].

In the present study, COVID-19 doubled the risk of mortality with AMI. The COVID-19 pandemic has significantly impacted the care and outcomes of patients with AMI. Studies have shown that COVID-19 infection is associated with higher in-hospital mortality in AMI patients, with factors such as a high GRACE score and COVID-19 positivity being independent predictors of mortality [29]. Prior studies have shown that troponin elevations in COVID-19 often reflect myocarditis, microvascular injury, or pulmonary embolism and largely serve to unmask preexisting coronary atherosclerosis rather than de novo plaque rupture. As laboratory data are not available in the NRD, we did not assess troponin levels in our cohort [29,30]. We identified requirements for vasopressor support (OR 2.12; 95% CI 2.05-2.19; p < 0.001) and use of an Impella device (OR 2.59; 95% CI 2.44-2.75; p < 0.001) as independent predictors of 30-day mortality. These interventions, ascertained via ICD-10-PCS codes in our multivariable logistic model, reflect severe cardiogenic shock and hemodynamic collapse, consistent with the known high mortality associated with mechanical circulatory support. These findings align with Basir et al., who reported that increasing vasopressor requirement is associated with increased risk of mortality in patients presenting with AMI [31]. This finding underscores the impact of SARS-CoV-2 infection in unmasking high-risk myocarditis, endothelial dysfunction, and plaque rupture.

Chronic obstructive pulmonary disease (COPD) (OR 0.93), chronic kidney disease (CKD) (OR 0.97), hypertension (OR 0.95), dyslipidemia (OR 0.89), tobacco use (OR 0.88), and obesity (OR 0.90) showed ORs <1, a counterintuitive finding likely due to under-coding in rapidly fatal cases, survivor bias, and residual confounding inherent to administrative data.

Limitations

There are some limitations of the study as well that should be considered while interpreting the findings of the study. This study used a retrospective observational study design, which introduces inherent limitations and limits the ability to establish causal relationships between the observed predictors and mortality outcomes. The associations identified may be influenced by confounding factors that cannot be fully controlled. While the NRD offers broad insights into patient demographics, comorbidities, and hospital characteristics, it lacks detailed clinical data such as laboratory results, imaging findings, and granular information on clinical severity beyond the APR-DRG classification. This limitation restricts the ability to adjust for the nuanced clinical status of patients. The study period (January 1, 2020, to December 31, 2020) coincided with the onset of the COVID-19 pandemic. The pandemic may have introduced unique challenges in hospital admission patterns and treatment protocols. Key variables such as patient health behaviors, social determinants of health (e.g., education, living situation, social support), pre-hospital care differences (emergency medical services (EMS) response times, bystander cardiopulmonary resuscitation (CPR)), and in-hospital treatment nuances (door-to-balloon times, medication adherence) are not captured in the NRD and may confound observed relationships. Procedures (e.g., PCI, Impella placement) and diagnoses (e.g., heart failure, AKI) may be under- or over-reported. Patients who suffered out-of-hospital cardiac arrest and died before hospital arrival, or those managed at non-participating or federal hospitals (e.g., Veterans Affairs (VA) facilities), are not represented, potentially biasing mortality estimates.

## Conclusions

In conclusion, this study identified multiple important predictors of 30-day mortality in AMI patients. Advanced age emerged as a key risk factor, while younger patients experienced noticeably better survival. Male sex was associated with a modestly higher risk. Medicare coverage and lower income appeared linked to worse outcomes, likely reflecting underlying age and socioeconomic challenges rather than direct effects. Clinical complications such as cardiac arrest, respiratory failure, liver failure, shock, and gastrointestinal bleeding were all markers of substantially increased mortality risk. By contrast, patients who underwent guideline-recommended revascularization procedures (PCI or coronary artery bypass grafting) or who had certain comorbidities like obesity or hypertension exhibited more favorable outcomes, which may reflect both therapeutic benefit and selection biases. Concurrent COVID-19 infection markedly worsened prognosis, highlighting its role in unmasking vulnerable coronary disease. Hospital characteristics, including ownership model, bed size, and urban versus micropolitan setting, also influenced survival. Future work should focus on longitudinal studies, linkage of administrative and clinical registry data, evaluation of COVID-19 vaccination impacts, and system-level interventions designed to reduce AMI mortality and address persistent health disparities.
